# The effect of myocardial fibrosis on left ventricular diastolic function assessed by non-invasive cardiac magnetic resonance and echocardiography

**DOI:** 10.1186/1532-429X-14-S1-O37

**Published:** 2012-02-01

**Authors:** Zahra Alizadeh Sani, Azarakhsh Babolian, Pedram Golnari

**Affiliations:** 1 Shaheed Rajaie Cardiovascular Medical & Research Center, Tehran University of Medical Sciences, Tehran, Islamic Republic of Iran

## Background

Accumulating evidence indicates that myocardial fibrosis contributes to the pathogenesis of diastolic dysfunction. However, investigating this issue has long been hampered by lack of suitable methodology. We aimed to define the effect of fibrosis on left ventricular diastolic function, using cardiac magnetic resonance (CMR) and Doppler echocardiography.

## Methods

Ninety one eligible subjects undergoing cardiac structural and functional assessment with LGE-CMR and Doppler echocardiography were investigated. Left ventricular function, volumes, myocardial thickness, left atrial volume, wall motion score index (WMSI) and the extent of LGE were assessed using CMR. Mitral early diastolic peak (Ewave) and late peak (A-wave) velocities, E/A ratio, deceleration time (DT) of mitral early velocity, early diastolic mitral annulus peak velocity was measured by echocardiography, and ratio of transmitral diastolic peak velocity to the mitral annular diastolic peak velocity (E/E’) was calculated. Classification of diastolic function as normal, mild dysfunction (impaired relaxation), moderate dysfunction (pseudonormal), and severe dysfunction (restrictive filling pattern) was performed according to standard criteria. The E/E ratio was used to estimate LV filling pressures.

## Results

The majority (90%) of subjects with normal diastolic function exhibited no or minimal fibrosis (median LGE score, 0; interquartile range, 0 to 0). The prevalence of LGE positivity by diastolic filling pattern was 11.8% in impaired relaxation, 100% in pseudonormal, and 75% in restrictive filling (P<0.001). LGE score significantly and progressively increased with increasing severity of filling impairment, reaching 5 (interquartile range, 0.5-9.75) in patients with restrictive filling pattern (P<0.001). LGE was observed in 80% of patients with ischemic cardiomyopathy and 26.3% of patients with nonischemic cardiomyopathy, but only in 3.6% of patients with repaired tetralogy of Fallot and none of patients with simple congenital heart disease (P<0.001). LGE score progressively increased with increasing left ventricular filling pressure estimated by tissue Doppler imaging-derived E/E (P<0.001, r=0.455). However it did not have any significant correlation with other echocardiographic diastolic function indices, such as E velocity, A velocity, and DT. Multiple linear regression showed LGE score (P=0.026, B =0.251) and WMSI (P<0.001, B=0.422) to be positively predictive for E/E value.

## Conclusions

Severity of myocardial fibrosis by LGE significantly correlates with the degree of diastolic dysfunction in a broad range of cardiac conditions, and among diastolic echocardiographic indices E/E may provide more valuable insight to this severity. Noninvasive assessment of myocardial fibrosis may provide valuable insights into the pathophysiology of left ventricular diastolic function and therapeutic response.

## Funding

This project was not funded.

